# Mean-field based framework for forward modeling of LFP and MEG signals

**DOI:** 10.3389/fncom.2022.968278

**Published:** 2022-10-13

**Authors:** Federico Tesler, Núria Tort-Colet, Damien Depannemaecker, Mallory Carlu, Alain Destexhe

**Affiliations:** CNRS, Paris-Saclay Institute of Neuroscience (NeuroPSI), Paris-Saclay University, Saclay, France

**Keywords:** magnetoencephalography (MEG), local field potential (LFP), mean field (MF), whole brain analysis, forward modeling

## Abstract

The use of mean-field models to describe the activity of large neuronal populations has become a very powerful tool for large-scale or whole brain simulations. However, the calculation of brain signals from mean-field models, such as the electric and magnetic fields, is still under development. Thus, the emergence of new methods for an accurate and efficient calculation of such brain signals is currently of great relevance. In this paper we propose a novel method to calculate the local field potentials (LFP) and magnetic fields from mean-field models. The calculation of LFP is done *via* a kernel method based on unitary LFP's (the LFP generated by a single axon) that was recently introduced for spiking-networks simulations and that we adapt here for mean-field models. The calculation of the magnetic field is based on current-dipole and volume-conductor models, where the secondary currents (due to the conducting extracellular medium) are estimated using the LFP calculated *via* the kernel method and the effects of medium-inhomogeneities are incorporated. We provide an example of the application of our method for the calculation of LFP and MEG under slow-waves of neuronal activity generated by a mean-field model of a network of Adaptive-Exponential Integrate-and-Fire (AdEx) neurons. We validate our method *via* comparison with results obtained from the corresponding spiking neuronal networks. Finally we provide an example of our method for whole brain simulations performed with The Virtual Brain (TVB), a recently developed tool for large scale simulations of the brain. Our method provides an efficient way of calculating electric and magnetic fields from mean-field models. This method exhibits a great potential for its application in large-scale or whole-brain simulations, where calculations *via* detailed biological models are not feasible.

## 1. Introduction

The electric and magnetic fields generated by neuronal currents are two commonly used brain signals to study neuronal activity. The measurement of these two signals involve different experimental techniques that can capture neuronal activity at different scales. The registration of electric local field potentials (LFP) involves invasive experimental techniques to measure the electric potential in the cerebral extracellular medium and it captures the activity of thousands of nearby neurons, in particular their synaptic activity (Niedermeyer and Lopes Da Silva, 2005; Buzsáki et al., 2012). On the other hand, the measurement of the magnetic field in MEG (magnetoencephalogram) is a non-invasive technique that captures the field generated by thousands to millions of neurons. Among non-invasive techniques to measure neuronal activity, MEG provides a relatively high spatial and temporal resolution, which are currently in the order of millimeters and milliseconds, respectively (De Pasquale et al., 2010; Hansen et al., 2010; Stokes et al., 2015).

The calculation of these signals from the underlying neuronal sources (usually referred to as “forward-modeling”) can be performed *via* detailed biophysical models (Hämäläinen et al., 1993; Bédard et al., 2004; Nunez et al., 2006; Bédard and Destexhe, 2012; Lindén et al., 2014; Hagen et al., 2018; Ilmoniemi and Sarvas, 2019). Although successful, this turns out to be very computationally demanding, specially when large populations of neurons are into consideration. Nowadays the utilization of mean-field models describing the activity of neuronal populations has become a powerful tool for large-scale simulations (Sanz Leon et al., 2013; Depannemaecker et al., 2021), however the calculation of brain signals from the mean-field models is still under development. Thus, the emergence of new modeling-frameworks that allow an accurate and efficient calculation of multiple brain-signals from mean-field models is currently of great interest.

In this paper we provide a method to calculate the LFP and MEG signals from mean-field models. To calculate the LFP we base our method on the unitary LFP (uLFP), which is the LFP generated by the post-synaptic currents from a single axon. We adopt a recently developed kernel-method based on uLFP's for spiking neural networks (Telenczuk et al., 2020) and we adapt this method for its application to mean-field models. Kernel methods based on neuronal models (Hagen et al., 2016) and experimental data (Telenczuk et al., 2020) have been proposed for the calculation of LFP's. In our approach we use a kernel method based on experimental data of uLFP's (Telenczuk et al., 2020), which is not restricted by specific model constraints.

To calculate the magnetic-field we make use of current-dipole and volume-conductor models for an inhomogeneous medium. We estimate the contribution of neuronal currents to the magnetic field from a mean-field model and we incorporate the previous estimation of the LFP to calculate the contribution of secondary currents due to the medium conduction. We provide an example of our method for the calculation of the LFP and MEG under slow-wave activity generated by a mean-field model of Adaptive-Exponential Integrate-and-Fire (AdEx) neurons. To validate our method we compare the results with the ones obtained from simulations of the corresponding spiking neural networks. Finally we provide an example of our method for whole brain simulations performed with The Virtual Brain (TVB), a recently developed tool for large scale simulations of the brain (Sanz Leon et al., 2013; Goldman et al., 2020).

## 2. Method

### 2.1. Calculation of the electric potential from mean-field models

To calculate the extracellular electric potential (LFP) we will make use of a recently developed kernel method based on unitary-LFP's (uLFP) (Telenczuk et al., 2020). This method was designed for the estimation of LFP's from spiking network simulations and consists in convolving the spikes of the network with two kernels, according to the formula:


(1) $$\begin{array}{l} V_{e}(\vec{x},t)={\int^{\;}}^{\;}K_{e}(\vec{x},t-\tau )(\sum_{j}\delta (\tau -t_{e,j}))d\tau \\ \;+{\int^{\;}}^{\;}K_{i}(\vec{x},t-\tau )(\sum_{j}\delta (\tau -t_{i,j}))d\tau \;, \end{array}$$

where $K_{e}\left(\vec{x},t-\tau \right)$ and $K_{i}\left(\vec{x},t-\tau \right)$ are the kernels associated with spikes from excitatory and inhibitory neurons, respectively, while {*t*_*e,j*_} and {*t*_*i,j*_} are the spiking times of excitatory and inhibitory neurons. In mean-field models, we typically deal with the mean firing activity of two populations, excitatory and inhibitory neurons, which we will note by ν_*e*_ and ν_*i*_. To adapt this method to mean-field models, the precise timing spikes in the previous expression can be replaced by the rates of spiking activity, ν_*e*_ and ν_*i*_, which yields:


(2) $$\begin{array}{l} V_{e}(\vec{x},t)=\int^{\;}K_{e}(\vec{x},t-\tau )\;\nu_{e}(\tau )\;d\tau \\ \;+\int^{\;}K_{i}(\vec{x},t-\tau )\;\nu_{i}(\tau )\;d\tau . \end{array}$$

where we have assumed that the spiking rates are high enough such that the typical inter-spike interval is small compared to the characteristic time of the kernel and thus the discrete spiking times can be replaced by the average firing rate. We notice that a typical size of a neuronal population in a mean-field is of thousands of cells with individual firing rates up to tens of Hz. Thus, for an electrode registering the activity of the population, this gives an inter-spike interval in the order of 1*x*10^−2^ ms, while the kernels (as shown below) have characteristic times of a few ms, which validates the previous assumption.

The kernels functions *K*_*e*_ and *K*_*i*_ were estimated from experimental data, by fitting a Gaussian template to the relation between single spikes and LFP's obtained in micro-electrode recordings (i.e., unitary LFP's) (Teleńczuk et al., 2017; Telenczuk et al., 2020). The kernels at position $\vec{x}$ and time *t* are given by:


(3) $$\begin{array}{l} K\left(\vec{x},t\right)=A\left(\vec{x}\right)\text{ exp}\left[-{\left(t-t_{p}\right)}^{2}/\left(2\sigma^{2}\right)\right], \end{array}$$

where *A* is the amplitude (which can be negative), σ is the standard deviation of the kernel in time, and *t*_*p*_ is the peak time of the kernel. The latter is given by


(4) $$\begin{array}{l} t_{p}=t_{0}+d+|\vec{x}-{\vec{x}}_{0}|/v_{a}, \end{array}$$

where *t*_0_ is the time of the spike of the cell, $\left|\vec{x}-{\vec{x}}_{0}\right|$ is the distance between position of the electrode $\vec{x}$ and the position ${\vec{x}}_{0}$ of the current source, *d* is a constant delay, and *v*_*a*_ is the axonal speed. We use the value of *v*_*a*_ = 200 mm/s, estimated from human LFP recordings (Teleńczuk et al., 2017).

**Figure 1:**
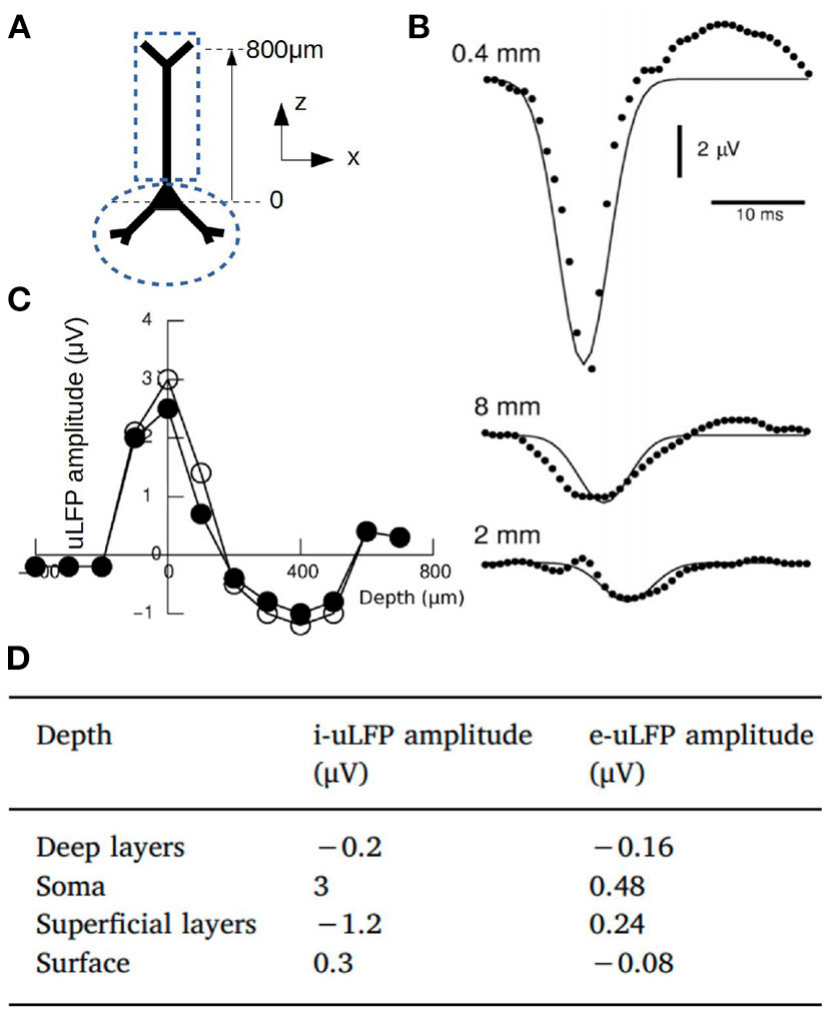
**(A)** Diagram of a pyramidal neuron and the coordinate system. The dashed lines indicate the two compartments considered for the calculation of the magnetic fields. **(B)** Experimental measurements of the uLFP (circles) at different distances from the soma (x-coordinate) together with the uLFP kernel of Equation (3) (solid line). **(C)** Numerically estimated uLFP amplitude as a function of depth (z-coordinate) for inhibitory neurons (white and black circles correspond to different simulations of the same system, see Telenczuk et al., 2020). **(D)** Amplitude of the uLFP for inhibitory and excitatory neurons at selected depths: Deep layers (*z* = –400 μm), Soma (*z* = 0),Superficial layers (*z* = 400 μm) and Surface (*z* = 800 μm). Adapted from Telenczuk et al. (2020).

To model the observed near-exponential amplitude decay with distance, the following expression can be used for $A\left(\vec{x}\right)$, in cylindrical coordinates (Teleńczuk et al., 2017):


(5) $$\begin{array}{l} A\left(\vec{x}\right)=A_{0}\left(z\right)\text{ exp}\left[-|x-x_{0}|/\lambda \right], \end{array}$$

where *A*_0_(*z*) is the maximal amplitude, which depends on coordinate *z* (cortical depth), |*x* − *x*_0_| is the radial distance between position of the electrode $\vec{x}$ and the source at ${\vec{x}}_{0}$ (see coordinate system in Figure 1 for a cylindrical region) and λ is the space constant of the decay. From human microelectrode recordings, λ was consistently found around 200–250 μm (Teleńczuk et al., 2017). A diagram of the kernel and the corresponding values for *A*_0_(*z*) are shown in Figure 1.

To perform the calculation of the LFP from mean-field models we will replace the single neuron magnitudes in the previous expressions by their mean values over the neuronal population. For the spatial dependence we take the average value of the function $A\left(\vec{x}\right)$ over a circular region around the point $\vec{x}$ where the field is measured. Taking into account the exponential decay, we will assume that the radius of the region of interest around $\vec{x}$ (*r*_*region*_) is defined by the characteristic distance λ, and we will take *r*_*region*_ = 2λ. For the temporal part of the kernel we will neglect the term corresponding to the axonal propagation, which holds valid as far as the propagation time is small compared with the typical temporal scale of the kernel, for example in the case where the region comprised by the neuronal population is small enough, as we will show in the example provided in the Results section. Thus, we will take: $A\left(\vec{x}\right)\approx A_{0}\left(z\right)<\text{exp}\left[-|x-x_{0}|/\lambda \right]>{|}_{r=2\lambda }=A_{0}\left(z\right)\frac{1}{2}\left(1-\frac{3}{e^{2}}\right)\approx 0.3A_{0}\left(z\right)$ (independent of λ) and *t*_*p*_ = *d*, where ‘<>’ indicates the mean over the area defined by the radius *r*_*region*_ = 2λ.

### 2.2. Calculation of the magnetic field from mean-field models

To estimate the magnetic field generated by a neuronal population we will assume that the variations in charge and current densities are slow enough (frequencies below the thousands of hertz) such that time derivatives in Maxwell equations can be neglected. Within this quasi-static approximation, the magnetic field generated by a current density *J*(*r, t*) is given by (Geselowitz, 1970; Hagen et al., 2018; Garcia-Rodriguez and Destexhe, 2021):


(6) $$B(r,t)\approx \frac{\mu_{0}}{4\pi }\int^{\;}J(r^{\prime },t)\times \frac{r-r^{\prime }}{|r-r^{\prime }{|}^{3}}{\text{d}}^{3}r^{\prime }$$

where μ_0_ is the magnetic susceptibility, *r*′ is the location of the source and *r* the point where the field is measured. For the case where $\left|r-r_{o}^{\prime }|>>|r^{\prime }-r_{o}^{\prime }\right|$, where $r_{o}^{\prime }$ represents the location of the center of the neuronal population we can take the far-field approximation and we have:


(7) $$\begin{array}{l} B(r,t)\approx \frac{\mu_{0}}{4\pi }Q\times \frac{r-r_{o}^{\prime }}{|r-r_{o}^{\prime }{|}^{3}}. \end{array}$$

where *Q* = ∫***J***(***r***′, *t*)d^3^*r*′ is the total dipole moment. This is in particular valid for the case of MEG, where sensors are located outside the scalp, far from the neuronal population generating the field.

In the brain, neurons are surrounded by the extracellular medium which is an electrical conductor. The current *J* is then composed of three main sources: synaptic currents (*J*^*s*^), action potentials (*J*^*a*^) and conduction currents (*J*^*c*^), the last ones being related to the induced currents in the conducting surrounding medium. Thus, we can write *Q*(*r, t*) = *Q*^*s*^ + *Q*^*a*^ + *Q*^*c*^. Currents related with action potentials tend to cancel each other, and their contribution to the magnetic field can be neglected (Lindén et al., 2014). In the following we show how to estimate the contributions from the conduction and synaptic currents. The conduction currents, given a medium conductivity σ(*r, t*), can be written as:


(8) $$\begin{array}{l} J^{c}=\sigma \left(r,t\right)E\left(r,t\right)=-\sigma \left(r,t\right)\nabla \Phi \end{array}$$

where *E*(*r, t*) is the electric field at point *r* and **Φ** is the local electric field potential (LFP). The conductivity σ(*r, t*) will in general depend on the position and may vary with time. We will compartmentalize the brain or region under study into small volume domains of constant conductivity, such that we can write for *Q*^*c*^ (Geselowitz, 1970; De Munck et al., 2012):


(9) $$\begin{array}{l} Q^{c}=-\int \sigma (r^{\prime },t)\nabla \Phi (r^{\prime },t)d^{3}r^{\prime }\\ \;=-\sum_{i}(\sigma_{i}{\left(t\right)}^{\prime }-\sigma_{i}{\left(t\right)}^{\prime \prime })\int \Phi (r^{\prime },t)dS_{i^{\prime }} \end{array}$$

where the summation goes over all surfaces of discontinuity, $\sigma_{i}{\left(t\right)}^{\prime }$ and $\sigma_{i}{\left(t\right)}^{\prime \prime }$ are the conductivities at the two regions separated by the surface *i*, *ds*′ is the differential area element for surface *i* and where the identity $\int_{V_{i}}\nabla^{\prime }\Phi (r^{\prime },t)d^{3}r^{\prime }=\int_{S_{\left(i\right)}}\Phi (r^{\prime },t)d{S^{\prime }}_{i}$ was used. We notice that the electric field potential Φ can be estimated from the mean-field models using the kernel method described in the previous section, from where the contribution of *Q*^*c*^ to the magnetic field is fully determined. We notice in addition that the conductivity σ may also depend on the frequency and direction. For simplicity in this paper we will assume an isotropic medium and independent of the frequency, but in principle these dependencies can be incorporated within our method.

To estimate the contributions of intrinsic neuronal currents to the MEG we will take into account that axial currents are believed to be the main source for the magnetic-field (in opposition to transmembrane currents) (Hämäläinen et al., 1993). We notice however that, in general, the mean-field and AdEx models are based on point-neurons (i.e., a single-compartment neuronal model). In order to incorporate axial currents to our model we will extend our description and consider the mean-field model in combination with a two compartment model (sometimes referred to as an “hybrid modeling”; Hagen et al., 2018), which is the minimum configuration that incorporates intra-neuron current flow. We show the diagram of the two-compartment model in Figure 1A. One of the compartments corresponds to the soma-perisomatic region and the other to the apical dendrites. We will assume that the membrane voltage for each of these compartment is given by:


(10) $$\begin{array}{l} C_{1}\frac{dV_{1}}{dt}=g_{L1}(E_{L}-V_{1})+I_{syn}^{1}-W+I_{A}\\ C_{2}\frac{dV_{2}}{dt}=g_{L2}(E_{L}-V_{2})+I_{syn}^{2}-I_{A} \end{array}$$

where *g*_*L*_ and *E*_*L*_ are the conductance and reversal potential of the leakage channel, $I_{syn}^{j}$ is the synaptic input to compartment *j*, *W* is a variable describing neuronal adaptation (which we only consider affecting the first compartment for simplicity) and *I*_*A*_ is the axial current between the two compartments, i.e., *I*_*A*_ = (*V*_1_ − *V*_2_)/*R*_*A*_, being *R*_*A*_ the axial resistance.

Following Kuhn et al. (2004), we will assume that the mean membrane potential of each compartment can be obtained by taking the stationary solution of (Equation 10) to static synaptic currents given by the synaptic bombardment with firing rates ν_*e*_ and ν_*i*_ (Di Volo et al., 2019). Thus, subtracting both expressions in Equation 10, and taking the stationary solution we get (for g_L1_ = g_L2_ = g_L_):


(11) $$\begin{array}{l} \mu_{V1}-\mu_{V2}=\frac{I_{syn}^{1}-I_{syn}^{2}-W}{2/R_{A}+g_{L}} \end{array}$$

where μ_*Vj*_ indicates the mean voltage of compartment *j* and from where the mean axial current (over the neuronal population) can be estimated. We notice that in general the synaptic currents will be a function of the voltage of each compartment and the firing rates of the population, in which case both stationary solutions of Equation 10 have to be considered. In a very general way we can write $I_{syn}^{j}=I_{syn_{ex}}^{j}+I_{syn_{in}}^{j}$, with $I_{syn_{ex}}^{j}$,$I_{syn_{in}}^{j}$ the excitatotory and inhibitory synaptic currents, respectively, given by:


(12) $$\begin{array}{c} I_{syn_{ex}}^{j}=K_{e}^{j}\mu_{G_{e}}\left(E_{e}-\mu_{Vj}\right)\\ I_{syn_{in}}^{j}=K_{i}^{j}\mu_{G_{i}}\left(E_{i}-\mu_{Vj}\right) \end{array}$$

where μ_*G*_*e*(*i*)__ is the mean excitatory (inhibitory) synaptic conductance and $K_{e\left(i\right)}^{j}$ is the number of excitatory (inhibitory) synaptic connections arriving to compartment *j* (Di Volo et al., 2019). The estimation of the mean synaptic conductances is to be defined from the mean-field model of use. In the examples presented in this paper we will consider a conductance based network, where the conductance at each synaptic terminal is increased by a quantal amount *q*_*e*(*i*)_ for each pre-synaptic spike (see Section 3 for details). For these networks and assuming that the spiking activity follows a poissonian distribution, then the mean conductances can be written as (Di Volo et al., 2019):


(13) $$\begin{array}{l} \mu_{G_{e\left(i\right)}}=\nu_{e\left(i\right)}\tau_{e\left(i\right)}q_{e\left(i\right)} \end{array}$$

where τ_*e*(*i*)_ is the characteristic decay time of the synaptic conductance. With the definition of the synaptic currents and mean conductances, Equations (10) and (11) are fully determined and the axial currents can be calculated (see Supplementary material for further details). By further assuming that excitatory neurons are the main source of the magnetic fields then we have:


(14) $$\begin{array}{l} Q^{s}=n_{e}LI_{A}^{e} \end{array}$$

which gives the mean dipole moment generated by intrinsic neuronal currents, where *L* is a characteristic length of the dipole and *n*_*e*_ is the number of excitatory neurons. Thus, together with Equation (9) (*Q*^*c*^) and (7) the magnetic field is completely determined.

In the Results section we will present an example of the use of the method for a recently developed mean-field model of a network of Adaptive-exponential-Integrate-and-Fire (AdEx) neurons.

### 2.3. The TVB simulation platform

The Virtual Brain simulator platform (TVB) provides an environment for large-scale brain simulations, where the brain is compartmentalized in macro-domains represented as nodes in a network. Each node in the network is described *via* a mean-field model. The connectivity among brain regions is provided *via* a brain connectome. The size of the compartmentalization can be defined by the user, depending on the characteristic of the study and computational resources. For our simulations we will consider a coarse-grain simulation where each node represents a different brain region. For more details on our simulations see the Section 3. For details on the TVB platform see Sanz Leon et al. (2013) and Melozzi et al. (2017).

## 3. Results

We present in the following an example of our method for the calculation of the Local Field Potential (LFP) and magnetic fields from a mean-field model. We will validate our method *via* comparison with results obtained from simulations of the corresponding spiking neural networks. The example we present is based on simulations of slow-waves of neuronal activity obtained with a mean-field model of Adaptive-Exponential Integrate-and-Fire neurons. Slow-waves (0.5-3.5 Hz, δ-band) are a key feature of neuronal activity during deep-sleep and can be experimental measured *via* LFP and MEG (Destexhe et al., 1999; Simon et al., 2000; Lopes da Silva, 2013).

### 3.1. AdEx mean-field model

To provide an example and validate our method we will consider a recently developed mean-field model of a network of inhibitory and excitatory Adaptive-Exponential-Integrate-and-Fire (AdEx) neurons (Brette and Gerstner, 2005). The spiking AdEx neuronal model is defined by the system of equations:


(15) $$\begin{array}{l} C\frac{dV}{dt}=g_{L}\left(E_{L}-V\right)+g_{L}\Delta \;\text{exp}\left(\frac{V-V_{T}}{\Delta_{T}}\right)-w+I_{syn} \end{array}$$

(16) $$\begin{array}{l} \frac{dw}{dt}=b\sum \delta \left(t-t_{sp}\right)+\frac{a}{\tau_{w}}\left(V-E_{L}\right)-\frac{w}{\tau_{w}} \end{array}$$

where *V* is the membrane potential, *w* is an adaptation current, *C* = 200 pF is the membrane capacity, *g*_*L*_ = 10 nS is the leakage conductance, *E*_*L*_ = −63 mV is the leakage reversal potential, *V*_*T*_ = −50 mV, Δ = 2 mV (0.5 mV) for excitatory (inhibitory) neurons, *I*_*syn*_ is the synaptic current, *a* is the sub-threshold adaptation constant and *b* is the spiking adaptation constant. When *V* > *V*_*T*_ at time *t* = *t*_*sp*_ a spike is generated, the membrane potential is reset to *V*_*res*_ = −65 mV and remains at this value for a refractory time *t*_*ref*_ = 5 ms, and the adaptation variable is increased by an amount *b*. In our simulations we will consider that no adaptation occurs in inhibitory neurons (i.e., *a* = *b* = 0), while for excitatory neurons we will consider *b* = 60 pA and *a* = 0. The synaptic current *I*_*syn*_ received by a neuron is given by the spiking activity of all presynaptic neurons connected to it. The total synaptic current can be written as a sum of the excitatory and inhibitory synaptic activity *I*_*syn*_ = *G*_*e*_(*E*_*e*_ − *V*) + *G*_*i*_(*E*_*i*_ − *V*), where *E*_*e*_ = 0 (*E*_*i*_ = −80 mV) is the excitatory (inhibitory) reversal potential and *G*_*e*_, *G*_*i*_ are the synaptic conductances. We model the synaptic conductances as decaying exponential functions that experience a quantal increase *q*_*e*_ and *q*_*i*_ at each pre-synaptic spike: $G_{e\left(i\right)}=q_{e\left(i\right)}\sum \Theta \left(t-t_{sp}\right)e^{\frac{t-t_{sp}}{\tau_{e\left(i\right)}}}$, where *q*_*e*_ = 1.5 nS, *q*_*i*_ = 5 nS, τ_*e*_ = τ_*i*_ = 5 ms.

We will consider a network of 10,000 neurons, with 80% of excitatory and 20% of inhibitory neurons. Neurons in the network are randomly connected with probability *p* = 5%.

The mean-field equations for the AdEx network are given to a first-order by (Di Volo et al., 2019):


(17) $$\begin{array}{l} T\frac{d\nu_{e,i}}{dt}=F_{e,i}(W,{\bar{\nu }}_{e},\nu_{i})-\nu_{e,i}\\ \frac{dW}{dt}=-\frac{W}{\tau_{w}}+b\nu_{e}+a(\mu_{V}({\bar{\nu }}_{e},\nu_{i},W)-E_{L}) \end{array}$$

where ν_*e,i*_ is the mean neuronal firing rate of the excitatory and inhibitory population, respectively, *W* is the mean value of the adaptation variable, *F* is the neuron transfer function (i.e., output firing rate of a neuron when receiving excitatory and inhibitory inputs with mean rates ν_*e*_ and ν_*i*_ and with a level of adaptation W), *a* and *b* are the sub-threshold and spiking adaptation constants, *t*_*w*_ is the characteristic time of the adaptation variable, *T* is a characteristic time for neuronal response, μ_*V*_ is the average membrane voltage and *E*_*L*_ is the leakage reversal potential (see Di Volo et al., 2019 for details).

### 3.2. Two compartment AdEx model

To validate the results of our method for the calculation of magnetic fields, we will compare the estimation from our method with the results obtained from a two-compartment spiking AdEx network. In this network inhibitory neurons will be consider as point neurons as before, while excitatory neurons will be described by a two-compartment model in analogy to the model proposed in Equation (10). The equations corresponding to the peri-somatic area (Equations 18 and 19) will take into account the spiking mechanism and the adaptation. We will consider that the soma receives inputs from synaptic currents and from the axial current coming from the dendritic region:


(18) $$\begin{array}{l} C_{S}\frac{dV_{S}}{dt}=g_{L_{S}}\left(E_{L}-V\right)+g_{L_{S}}\Delta_{T}\text{exp}\left(\frac{V-V_{T}}{\Delta_{T}}\right)-w+I_{A}+I_{syn-S} \end{array}$$

(19) $$\begin{array}{l} \frac{dw}{dt}=b\sum \delta \left(t-t_{sp}\right)+\frac{a}{\tau_{w}}\left(V_{S}-E_{L}\right)-\frac{w}{t_{w}} \end{array}$$

Where *C*_*S*_ = 200 pF, *g*_*LS*_ = 10 nS, *E*_*L*_ = −63 mV, *V*_*T*_ = −50 mV, Δ_*T*_ = 2 mV, τ_*w*_ = 500 ms, *b* = 60 pA *a* = 0.

The axial current is defined as before *I*_*A*_ = *g*_*A*_(*V*_*D*_−*V*_*S*_) with *g*_*A*_ = 400*nS*.

The dendritic membrane potential is described by a leak current and receives input from synaptic currents. The membrane voltage of the dendrite is described by:


(20) $$\begin{array}{l} C_{D}\frac{dV_{D}}{dt}=g_{LD}\left(E_{L}-V_{D}\right)-I_{A}+I_{syn-D} \end{array}$$

With *C*_*D*_ = 10 pF, and *g*_*L*−*D*_ = 2 nS.

For both populations when an action potential is emitted (voltage is greater than *V*_*T*_), the system is reset to its resting value and remains at that value for a refractory period of *t*_*ref*_ = 5 ms. We will take *V*_*T*_ = −40 mV, *V*_*R*_ = −55 mV for excitatory cells and *V*_*T*_ = −47.5 mV, *V*_*R*_ = −65 mV for inhibitory cells.

To estimate the magnetic field from the spiking network we will take the mean axial current generated in the excitatory neurons of the network and proceed to calculate the mean dipole moment as in Equation (14).

### 3.3. LFP from the AdEX mean-field model

We provide next two examples of the method to calculate the LFP. First we show the results for a single mean-field model of an AdEx network, and the second example consists in a large-scale simulation of the human brain performed with The Virtual Brain simulator platform.

**Figure 2:**
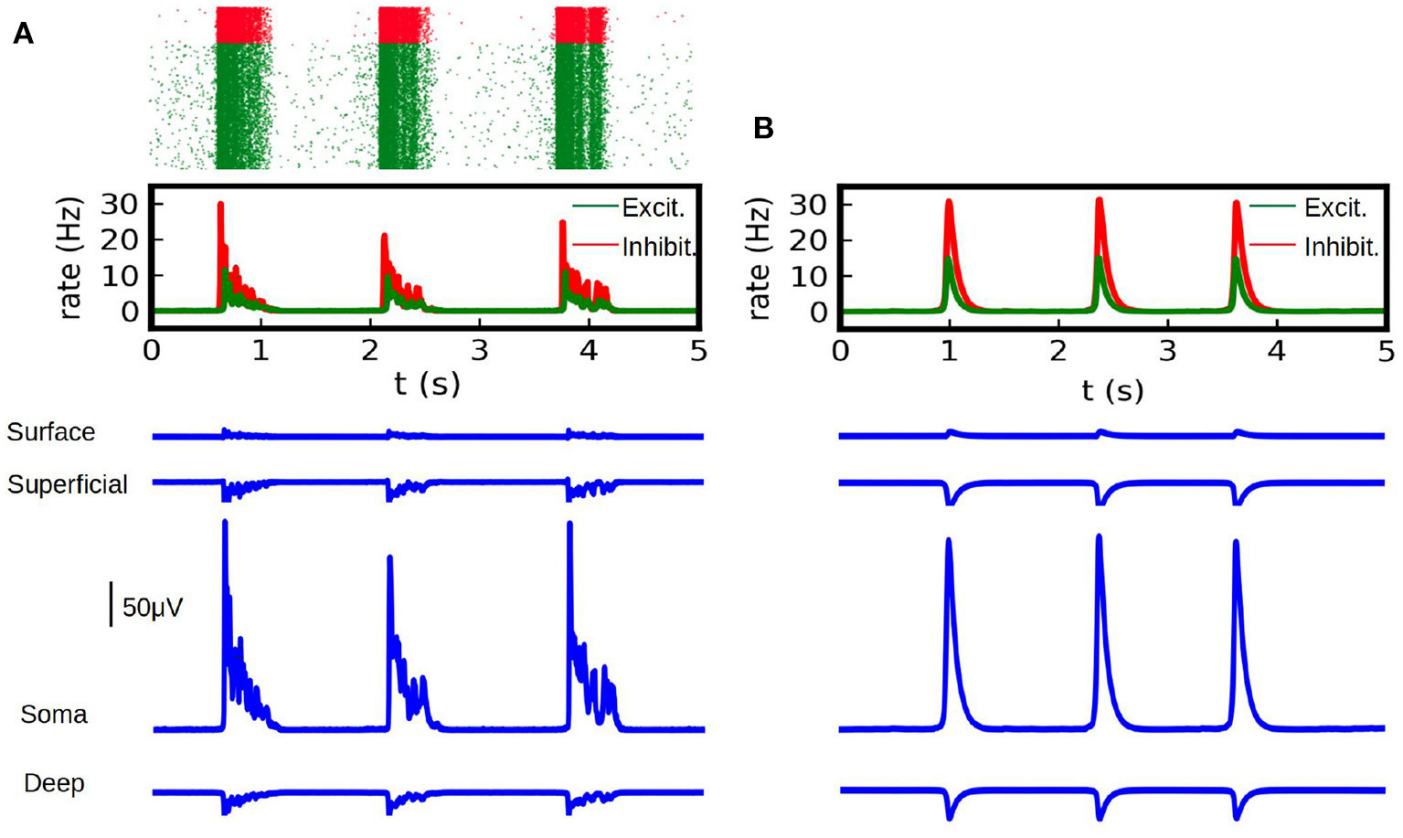
**(A)** LFP calculations obtained with the kernel method on spiking-network simulations (in blue) at different depths (Surface, Superficial, Soma and Deep, see table in Figure 1). Simulations were performed on a network of 10,000 AdEx neurons and LFP was calculated according to Equation (1). Neurons where uniformly distributed over a square surface of 1 x 1 mm^2^ (see diagram in Figure 4C for reference). We show on the top the raster plot of the neuronal activity during the simulation, for excitatory (green) and inhibitory (red) neurons together with the corresponding firing rate for each population. **(B)** LFP calculations obtained with the kernel method from mean-field simulations. We show the average firing rate for the excitatory and inhibitory neurons (top) in a network of 10,000 AdEx neurons, computed from Equation (17) and the corresponding LFP obtained with Equation (2) (blue).

In Figure 2 we show the results of the mean-field model and the LFP calculations for the first example. To validate our results we show the comparison with the calculation of the LFP for the corresponding spiking AdEx network. For the latter case we show the raster plot and the corresponding mean firing rate of the excitatory and inhibitory populations. We can see that the results from the mean-field model can correctly capture the amplitude and duration of the up-and-down states obtained in the LFP from spiking-networks. The average amplitude of the high-state in the LFP is of (206 ± 26) μV for the spiking case and (209 ± 2) μV for the mean-field case. The average duration of the high-state in the LFP is (0.59 ± 0.14) s for the method applied to the spiking network and (0.57 ± 0.09) s for the mean-field case. The duration is measured as the time between the beginning and the end of the high state, where the criteria for the starting and ending points is defined as the crossing point of 3 standard deviations from the base state.

**Figure 3:**
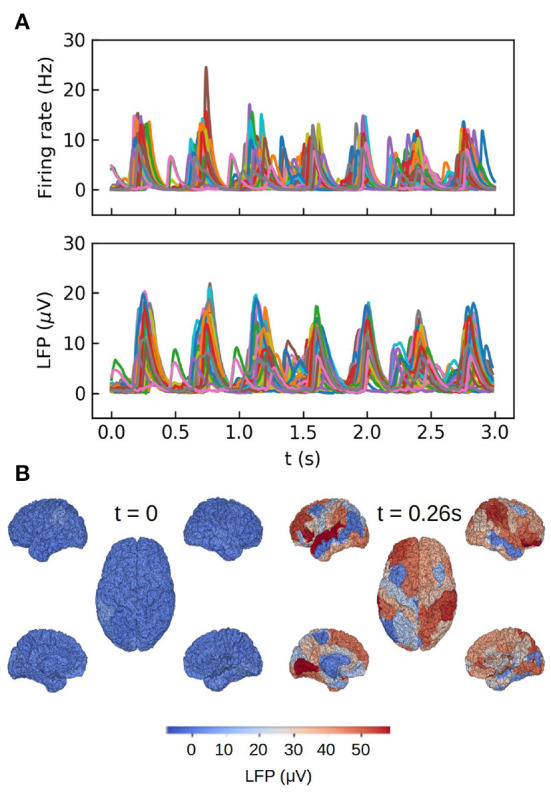
Whole brain simulations of the human brain performed with The Virtual Brain (TVB) platform. **(A)** Top: time course of the average firing rates for each brain region in the simulations. Bottom: Local field potential calculated with the kernel method for each region. **(B)** Colormap of the LFP amplitude at each brain region for a down (*t* = 0) and up (*t* = 0.26 s) states during one slow-wave oscillation. For details on the TVB simulator of the human brain see Sanz Leon et al. (2013), Schirner et al. (2015), Sanz-Leon et al. (2015), and Goldman et al. (2021).

The second example we present consists in a large-scale simulation of the human brain, performed with the TVB simulator platform. In this simulation each region of the brain is represented by one neuronal ensemble of AdEx neurons (as described in the first example). The connection between regions was defined by human tractography data (https://zenodo.org/record/4263723,Berlinsubjects/QL_20120814) from the Berlin empirical data processing pipeline (Schirner et al., 2015; Goldman et al., 2021). A parcellation of 68 regions is used, with long-range excitatory connections and delays defined by 120 tract length and weight estimates in human diffusion magnetic resonance imaging (dMRI) data (Sanz-Leon et al., 2015; Goldman et al., 2021). The LFP is calculated using the kernel-method, for which it has been incorporated in the TVB platform. For details on the connectome and the simulations with the TVB see Sanz Leon et al. (2013), Schirner et al. (2015), Sanz-Leon et al. (2015), and Goldman et al. (2021). The results are shown in Figure 3, where we show only the LFP for the Surface depth. All the simulations presented in the paper were performed using the python language and the TVB simulation platform for whole brain simulations.

### 3.4. Magnetic field from the AdEx mean-field model

In the following we proceed with the calculation of the magnetic field for the example of the AdEx mean-field model, as explained in the Section 2. As for the LFP, we consider two examples, one consisting in a single mean-field and another comprising a large-scale simulation of the human brain.

**Figure 4:**
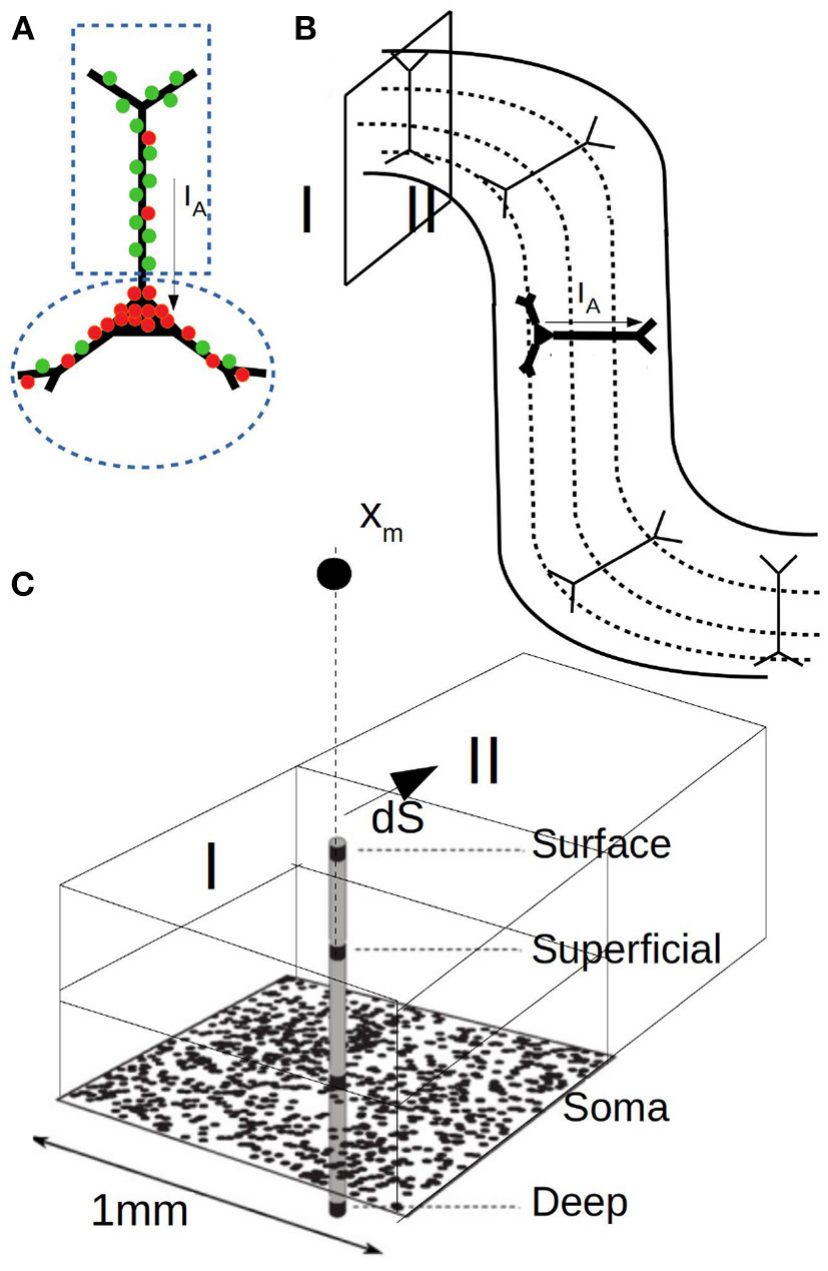
**(A)** Diagram of the two compartment neuronal model used for MEG calculations. One compartment covers the soma and perisomatic region with high concentration of inhibitory synapses (red) and the second compartment covers the apical dendrites with dominant concentration of excitatory synapses (green). **(B)** Diagram of the cerebral cortex with the arrangement and orientation of neurons in the gyrus and sulcus. The different depth layers used for LFP calculations are indicated by dashed lines. **(C)** Diagram of the domains, layers and surface consider for the MEG simulations. *X*_*m*_ indicates the position of the sensor.

To perform the calculations two points are to be specified: i) the synaptic input and ii) the specific geometry and neuronal distribution of the area under study. The first one is defined by the mean-field model adopted for the simulations while the second depends on the specific features of the study. In our case the synaptic currents are given by Equation (12) and (13). For our simulations we adopt τ_*e*(*i*)_ = 5ms, *q*_*e*(*i*)_ = 1.5nS (5 nS) and *E*_*e*(*i*)_ = 0 (−80 mv). For $K_{e\left(i\right)}^{j}$ we take $K_{e\left(i\right)}^{j}=n_{e\left(i\right)}p_{e\left(i\right)}^{j}$ with *n*_*e*(*i*)_ the number of excitatory (inhibitory) neurons in the population and $p_{e\left(i\right)}^{j}$ the connectivity probability of an excitatory (inhibitory) synapse at compartment *j*. To define $p_{e\left(i\right)}^{j}$ we assume as before that every two neurons in the network are connected with a probability *p*_*o*_ = 0.05. In addition, following experimental data, we will assume that inhibitory synapses have a higher concentration around the soma while excitatory synapses dominate in the apical dendrites (Megıas et al., 2001). We will consider a 60–40% distribution for inhibitory synapses (60% in the peri-somatic region, 40% at the apical dendrites) and a 30–70% distribution for excitatory synapses (see Figure 4A for a diagram of the two-compartment model). Thus, $p_{e\left(i\right)}^{1}=0.3p_{o}$ (0.6*p*_*o*_) and $p_{e\left(i\right)}^{2}=0.7p_{o}$ (0.4*p*_*o*_). Given the synaptic currents for each compartment, the axial current and its associated magnetic field is determined from Equation (7), (10), and (14).

To perform the simulations a concrete geometry and neuronal distribution for the region under study must be defined. For this, we will assume that neurons are aligned as shown in Figure 4B and uniformly distributed. It is easy to see that current dipoles oriented perpendicular to the scalp have a smaller or null contribution to the magnetic field and that the MEG is rather driven by dipoles oriented tangentially, which is a commonly accepted feature (Hämäläinen et al., 1993). For our simulations we will consider cubic regions as indicated in Figure 4C and for simplicity we will assume that only neurons and dipole moments with purely tangential currents (parallel to the scalp) contribute to the magnetic field. Furthermore, we will assume that the vector $r-r_{o}^{\prime }$ between the dipole and the sensor is aligned in the radial direction (i.e., perpendicular to the scalp), such that the calculated field corresponds to a purely tangential component (a generalization for an arbitrary angle is straightforward). We assume that the sensor is located at a distance of 3 cm from the neuronal population. For the contribution of the conduction currents (Equations 8 and 9) we will consider a single interface between two regions of different conductivity. In particular we will assume that the two conductivities correspond to the one of the gray matter and the cerebrospinal fluid, respectively, which exhibit a relatively high difference in conductivity (we use σ = 0.3 and 2.1 S/m, respectively, McCann et al., 2019; Mandija et al., 2021). The calculation of the LFP for Equation (9) and the related parameters follow the ones indicated for Figure 2.

**Figure 5:**
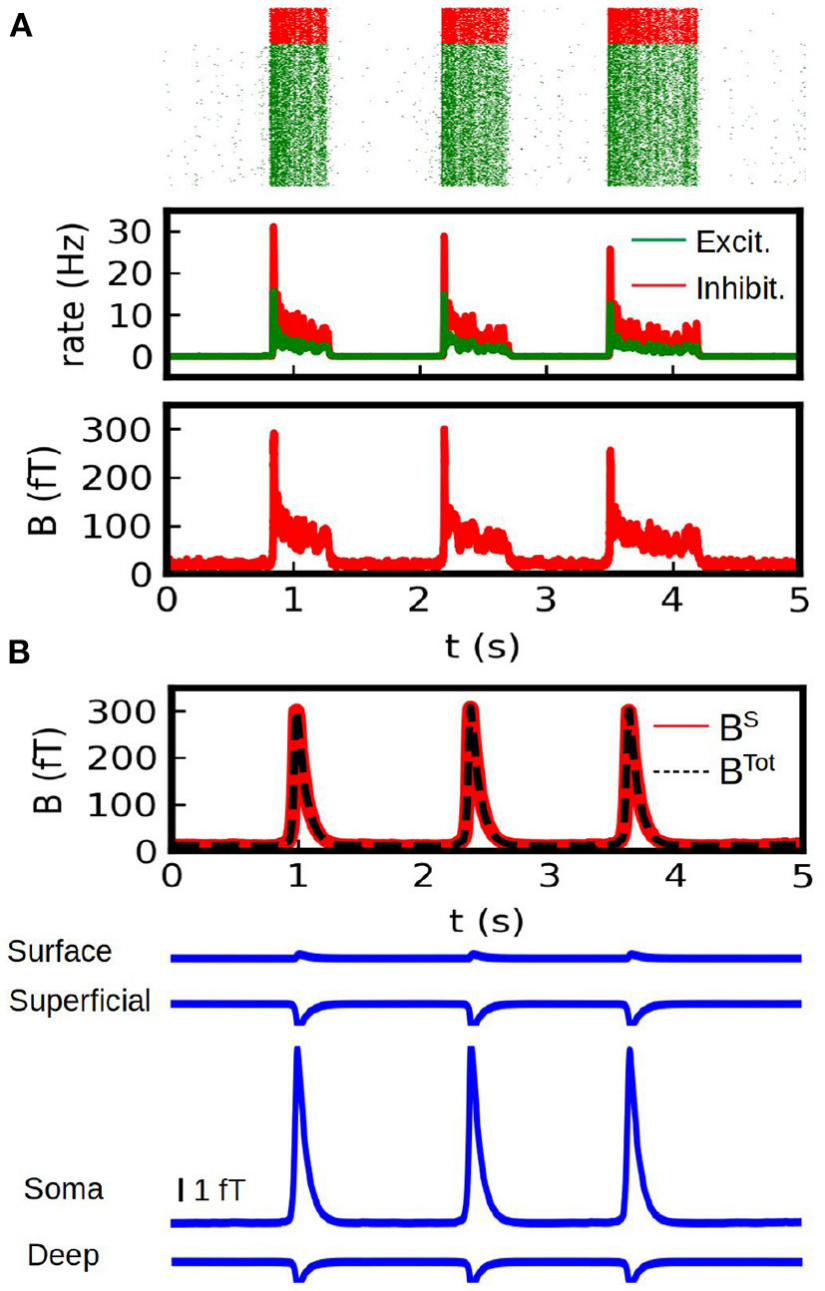
Calculation of the MEG signal. **(A)** Simulations from a spiking two-compartment AdEx network. We show the raster plot (top), the mean firing rate (middle) and the magnetic field generated by axional neuronal currents (bottom). **(B)** Results obtained with our method from the AdEx mean-field model. In the first plot (top) we show the total magnetic field generated by the system (axial neuronal currents and conduction currents, dashed black line) together with the contribution of the axial currents (solid red line). Bottom: Contribution to the magnetic field by of the conduction currents. The contribution of the different depths is shown.

In Figure 5B we show the results of our simulations. We show here the total magnetic field and the field generated by each different source (synaptic and conductance currents). For the conductance currents we show the contribution of each depth to the field. To validate our results we show the results obtained from the spiking AdEx two-compartment model in Figure 5A. For the spiking networks we show the raster plot, the mean firing rates and the resulting magnetic field. For this last one we only consider the contribution of synaptic (i.e., axial) currents to the field. The results from the mean-field method can reasonably capture the amplitude and duration of the up-and-down states obtained from the spiking network. The average amplitude of the up-state in the MEG is of (276 +/− 40) fT for the spiking case and (305 +/− 3) fT for the mean-field case. The average duration is of (0.87 +/− 0.43) s for the spiking case and (0.53 +/− 0.01) s for the mean-field case.

From these simulations we can see that the axial neuronal currents constitute the main source for the MEG, which is in agreement with typical experiments. In our simulations we took only a single interface between regions of different conductivity. The contribution of the different sources to the MEG will depends of course on the particular geometry, conductivities and neuronal distributions. Nevertheless, it is usually accepted that the magnetic field is mainly driven by axial neuronal currents (Hämäläinen et al., 1993). Our analysis would agree with this, but it also provides means to further analyze the different contributions and in particular to explore the possible contributions of different layers. For a comparison with experimentally measured slow-waves with MEG see for example Simon et al. (2000).

To end this section we present the results of the MEG calculations for a large-scale simulation of the human brain with The Virtual Brain platform, corresponding to the same set of simulations as in Figure 3. For the MEG we aim to describe the fields measured by *N* sensors and generated by the *M* sources (brain regions in our case). The magnetic field measured by a sensor at position *r* outside the conductor can be written in general as (Mosher et al., 1999 and Sanz-Leon et al., 2015):


(21) $$\begin{array}{l} B=G\left(r,r_{Q}\right)Q \end{array}$$

where ***Q*** indicates the corresponding dipole moments, ***r***_*Q*_ is the location of the sources (dipoles) and ***G***(***r, r***_*Q*_) is called the gain or transfer matrix and contains all the information regarding the geometry of the specific head-model under consideration. This is a generalization of Equation (7). In the TVB platform the gain matrix can be computed and registered for different head-models given the locations of sources and sensors (Sanz-Leon et al., 2015). In our simulations we will consider that each dipole moment is located at the center of the corresponding brain region and that there is one sensor per each brain region. For simplicity we will consider $G=\frac{\mu_{0}}{4\pi |r-r^{\prime }{|}^{2}}I$, where, as before, we assume that the vector ***r*** − ***r***′ is aligned in the radial direction, |***r*** − ***r***′| is a typical distance between the sensor and the source, and ***I*** is the identity matrix, such that each sensor measures only the field generated by each brain region. For a more detailed calculation of MEG, a more detailed head-model and orientation of dipoles should be taken into consideration.

**Figure 6:**
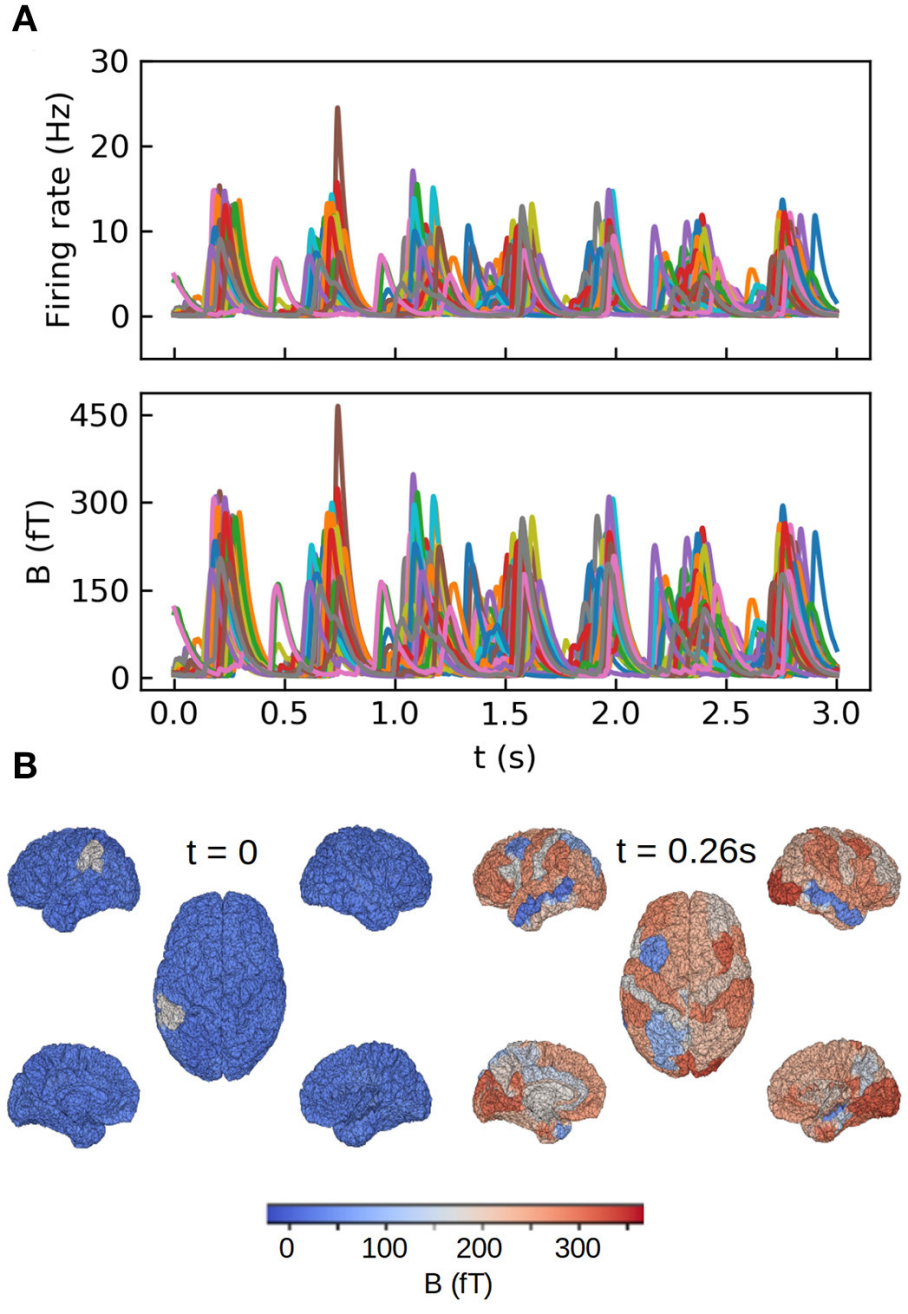
Whole brain simulations of the MEG signal in the human brain performed with The Virtual Brain (TVB) platform. **(A)** Top: time course of the mean firing rates for each brain region in the simulations. Bottom: magnetic field for each region calculated with the method introduced in this paper. **(B)** Colormap of the magnetic field amplitude at each brain region for a down (*t* = 0) and up (*t* = 0.26 s) states during one slow-wave oscillation. For details on the TVB simulations of the human brain see Sanz Leon et al. (2013), Schirner et al. (2015), Sanz-Leon et al. (2015), and Goldman et al. (2021).

We show in Figure 6 the results of our whole brain simulations. In Figure 6A of the figure we show the time series of the mean firing rates of each region and the corresponding magnetic field for each sensor registered in the simulation. For these simulations we are assuming an homogeneous medium (i.e., uniform conductivity), from where the only source of the magnetic field are the neuronal axial currents. In Figure 6B we show a colormap of the magnetic field for region corresponding to a single slow-wave in our simulations.

## 4. Discussion

In this paper we have introduced a method for forward modeling of local field potentials (LFP) and magnetic fields generated by neuronal activity. Our method allows to calculate these signals from mean-field models of neuronal populations. We have proposed a method based on a phenomenologically defined kernel to calculate the LFP, which provides an estimation of the field as a function of depth and distances from the cell body. To calculate the magnetic-field we utilized a current-dipole and volume conductor model which we combined with the kernel method of LFP to estimate the secondary currents induced in the conducting medium by the neuronal electric fields. Our method considers a very general scenario with a non-homogeneous conducting medium. Other efforts to calculate LFP signals from kernel methods have been previously proposed (Hagen et al., 2016; Skaar et al., 2020), where kernels are estimated from simulations of detailed biophysical neuronal models. Our method is based on experimentally measured uLFP's, which is not limited by specific model constraints.

We have presented an example of the application of our method for a mean-field of a population of Adaptive Exponential Integrate and Fire neurons. For the example shown in the paper the magnetic-field is mainly generated by neuronal currents (i.e., primary currents) with a nearly negligible contribution of the conduction currents (secondary currents).

One possible source of limitations of our method is associated with the characteristic of the mean-field models. In particular, the mean-field models presented here correspond to randomly connected networks, which prevents the utilization of specific network structures.

Finally, our method exhibits a great potential for application in large-scale or whole-brain simulations, where calculations *via* detailed biological models are not feasible. Further improvement of our method would include the refinement of the kernel used for the LFP calculations, which is envisioned for the near future. The specification of the kernel for different brain-regions and a more detailed analysis of its depth-dependence will lead to more accurate calculations of the local field potential and the MEG signal.

## Data availability statement

The raw data supporting the conclusions of this article will be made available by the authors, without undue reservation. The code for the implementation of the method introduced in this paper is available at https://doi.org/10.5281/zenodo.6983162.

## Author contributions

FT and AD designed the method. FT performed the simulations of the method for the mean-field models and analyzed the data. NT-C performed the simulations of the method for LFP in spiking networks. DD and MC designed and performed the simulations for the two-compartment spiking AdEx model. FT, DD, and AD wrote the manuscript. AD supervised the project. All authors contributed to the article and approved the submitted version.

## Funding

This research was supported by the CNRS and the European Union (Human Brain Project H2020-785907 and H2020-945539).

## Conflict of interest

The authors declare that the research was conducted in the absence of any commercial or financial relationships that could be construed as a potential conflict of interest.

## Publisher's note

All claims expressed in this article are solely those of the authors and do not necessarily represent those of their affiliated organizations, or those of the publisher, the editors and the reviewers. Any product that may be evaluated in this article, or claim that may be made by its manufacturer, is not guaranteed or endorsed by the publisher.
