# Supplementary material for: Mean-field based framework for forward modeling of LFP and MEG signals
Source: Front Comput Neurosci. 2022 Oct 13;16:968278. doi: 10.3389/fncom.2022.968278 (PMC9606720; doi:10.3389/fncom.2022.968278)
Supplement: Supplementary file 1 [file Data_Sheet_1.pdf]

Supplementary Material

Stationary solution for the two compartment model

The estimation of the mean neuronal axial current in our method is done by taking the stationary solution of the two-compartment model presented in Equation (10) of the main text. By equating both expressions to zero we get:

$$g_{L1}(E_L - V_1) + I_{syn}^1 - W + I_A = 0$$

$$g_{L2}(E_L - V_2) + I_{syn}^2 - I_A = 0 \qquad \text{(Eq. S1)}$$

In general $g_{L2} \neq g_{L1}$ and the synaptic currents typically depend on the voltage of each compartment. By using the expression of the synaptic currents given in Equation (12) of the main-text and solving for V_1 and V_2 in Eq. S1 we get:

$$V_1 = \frac{g_{L1} E_L + K_i^1 \mu_{Gi} E_i + K_e^1 \mu_{Ge} E_e - W + V_2 g_A}{g_{L1} + K_i^1 \mu_{Gi} + K_e^1 \mu_{Ge} + g_A}$$

$$V_2 = \frac{g_{L2} E_L + K_i^2 \mu_{Gi} E_i + K_e^2 \mu_{Ge} E_e + \frac{g_A D_1}{D_2}}{g_{L2} + K_i^2 \mu_{Gi} + g_A - \frac{g_A^2}{D_2}} \qquad \text{(Eq. S2)}$$

where g_A is the neuronal axial conductance, $D_1 = g_{L1} E_L + K_i^1 \mu_{Gi} E_i + K_e^1 \mu_{Ge} E_e - W$ and $D_2 = g_{L1} + K_i^1 \mu_{Gi} + K_e^1 \mu_{Ge} + g_A$. Here W is the adaptation variable. For the definition of the rest of parameters see the main-text.

From here the mean axial current is simply $I_A = g_A(V_2 - V_1)$. For the associated dipole moment see Equation (14) in the main-text.
